# Extensive *ARMC5* genetic variance in primary bilateral macronodular adrenal hyperplasia that started with exophthalmos: a case report

**DOI:** 10.1186/s13256-017-1529-3

**Published:** 2018-01-18

**Authors:** Ping Jin, Muhammad Usman Janjua, Qin Zhang, Chang-sheng Dong, Youbo Yang, Zhao-hui Mo

**Affiliations:** 1 Department of Endocrinology, The third Xiangya Hospital, Central South University, Tongzipo Road, Changsha, 410007 Hunan People’s Republic of China; 2 Department of Anesthesia, The affiliated Tumor Hospital of Xiangya Medical School of Central South University, Changsha, 410007 Hunan China

**Keywords:** PBMAH, Cushing’s syndrome, *ARMC5*, Exophthalmos

## Abstract

**Background:**

Primary bilateral macronodular adrenal hyperplasia is a rare cause of Cushing’s syndrome characterized by the presence of bilateral secretory adrenal nodules. Recent studies have shown that primary bilateral macronodular adrenal hyperplasia is caused by combined germline and somatic mutations of the *ARMC5* gene. Exophthalmos is an underappreciated sign of Cushing’s syndrome.

**Case presentation:**

A 52-year-old Chinese woman with progressively worsening bilateral proptosis presented to our hospital. Subsequently she was diagnosed as having primary bilateral macronodular adrenal hyperplasia and underwent bilateral laparoscopic adrenalectomy. Genomic deoxyribonucleic acid was isolated from lymphocytes as well as seven different adrenal nodules and the *ARMC5* sequence was determined by Sanger sequencing. We identified one heterozygous *ARMC5* germline mutation c.682C>T (p. Gln228*) and five heterozygous somatic mutations (c.310delG, c.347_357del11, c.267delC, c.283_289del7, and c.205-322del118) in five different adrenal nodules. All mutations are novel and were not found in any of the available online databases. To test whether the *ARMC5* mutation induced messenger ribonucleic acid decay, real-time reverse transcriptase polymerase chain reaction was performed on patient and control adrenal tissue. We found that the adrenal cortex of our patient showed a low *ARMC5* messenger ribonucleic acid expression compared with normal adrenal cortex, possibly as a result of nonsense-mediated messenger ribonucleic acid decay

**Conclusions:**

We demonstrated extensive genetic diversity of *ARMC5* in a patient with primary bilateral macronodular adrenal hyperplasia that started with exophthalmos, which contributes to further understanding of the pathogenesis of this disease. Early recognition of atypical symptoms and screening for *ARMC5* mutation in patients with primary bilateral macronodular adrenal hyperplasia has important clinical implications for the diagnosis and genetic counseling.

## Background

Primary bilateral macronodular adrenal hyperplasia (PBMAH), also known as adrenocorticotropic hormone (ACTH)-independent macronodular adrenal hyperplasia (AIMAH), is a rare cause of Cushing’s syndrome [1, 2], characterized by increased cortisol production from bilateral adrenal macronodules. Recently, the inactivating mutations of the armadillo repeat containing 5 (*ARMC5*, OMIM 615549) gene located at chromosome 16p11.2 were identified as a genetic cause of PBMAH [3, 4]. This gene has been proposed to acts as a tumor-suppressor gene where patients with PBMAH have a single germline mutation in *ARMC5*, acting as the first “hit,” and subsequent somatic mutation of the gene within adrenal tissue leading to the development of nodules and overproduction of cortisol [5–8]. In the current study, we present a case of a 52-year-old woman who presented to us with bilateral proptosis and was subsequently diagnosed as having PBMAH. *ARMC5* variations were analyzed in the deoxyribonucleic acid (DNA) of the lymphocytes and adrenal nodules of this patient.

This case report uncovers extensive genetic diversity of *ARMC5*, mutations which were not found in the available online database, and this case also highlights the importance of atypical symptoms of Cushing’s syndrome, such as exophthalmos, the early detection of which can prevent the patient from many detrimental effects of excessive cortisol exposure.

## Case presentation

A 52-year-old Chinese woman with progressively worsening bilateral proptosis presented to our hospital. She noticed bilateral proptosis 5 years ago. Although her thyroid tests were normal, she underwent retrobulbar injection of triamcinolone acetonide with no improvement of symptoms. She was diagnosed as having new onset of diabetes and hypertension during this time and was treated with insulin and amlodipine, respectively. Later she started developing proximal muscle weakness and was easily bruised respectively. She was a farmer belonging to a middle class family, living with her husband and two children, all healthy, in a remote city where she had lived for a major part of her life. She did not smoke tobacco or consume alcohol. She had no significant past medical history. She had not had any chronic illness or infectious disease previously. There was no family history of similar disease or any other chronic illness such as diabetes and hypertension. There was no history of allergy to any food or drugs. On physical examination: temperature (T) 36.5 °C, pulse (P) 72 beats/minute, respiration rate (R) 20 breaths/minute, blood pressure (BP) 140/90 mmHg, weight (W) 60 kg, and height (H) 156 cm. She showed typical Cushingoid appearance: skin atrophy, moon facies, buffalo hump, and purplish abdominal striae. She also had conjunctival congestion, edema, and lid retraction. Her respiratory movements were bilaterally symmetrical with a frequency of 20 breaths/minute. A cardiac examination was normal. S1 and S2 were normal with no added sounds. On abdominal examination no masses or tenderness were noted on both light and deep palpation. Her liver and spleen were not palpable. A sensory and motor system examination did not reveal any abnormality. Her neurological reflexes were normal. All cranial nerves were intact. Further ophthalmological examination revealed bilateral proptosis with Hertel’s exophthalmometer readings of 21 mm (right) and 22 mm (left). Intraocular pressure was elevated in her right eye (26 mmHg) and her left eye (27 mmHg). A fundus examination was normal. Extraocular movements were intact and bilateral visual fields showed no defect.

**Fig. 1:**
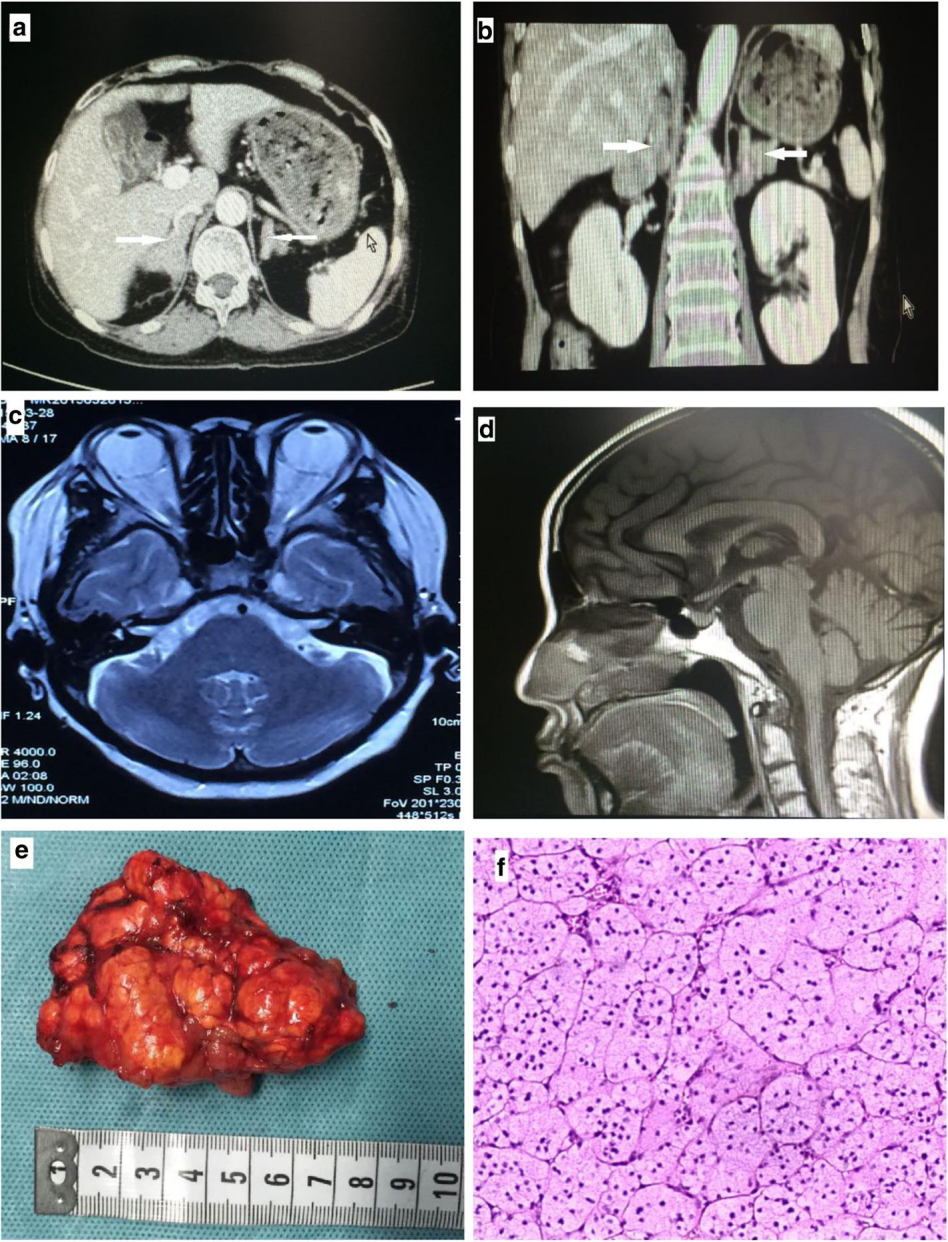
Clinical features of the patient with primary bilateral macronodular adrenal hyperplasia. Computed tomography scan of adrenal glands showed massive enlargement of the adrenal glands and multiple nodules (as shown by *arrows*) (**a**, **b**). Magnetic resonance imaging of the orbital showed bilateral exophthalmos (**c**). MRI of the pituitary gland was normal (**d**).Gross pathology of the resected left adrenal gland with nodules of various sizes (**e**). Histological view of resected adrenal tissue showing the presence of large cortical cells shown by hematoxylin and eosin stain at magnification × 100 (**f**)

Complete blood count, urine analysis, and renal and liver function tests were all normal. A biochemical evaluation revealed elevated 24-hour urinary free cortisol (UFC) 306.8 μg/24 hours (reference range 3.5 to 45 μg/24 hours), elevated cortisol level, and suppressed ACTH level with loss of their normal diurnal rhythm. Her cortisol level was 634 ug/L at 8 a.m., 621 ug/L at 4 p.m., and 521 ug/L at 0 a.m. (reference range 62 to 194 ug/L), while her ACTH level was always < 1 ng/L (reference range 7.2 to 63.6 ng/L). Serum cortisol remained unsuppressed after 1 mg overnight and high-dose dexamethasone suppression tests (at 624 ug/L and 607 ug/L, respectively). Serum thyroid function tests were normal; anti-thyrotropin receptor antibody (TRAb), anti-thyroid peroxidase (TPOAb), and anti-thyroglobulin antibodies (TGAb) were negative. Plasma potassium was 2.2~3.0 mmol/L. Glycated hemoglobin (HbA1c) was 8.7%. Serum androgen, aldosterone/renin activity ratio, and 24-hour urinary catecholamines were all in the normal range. Glucagon, luteinizing hormone-releasing hormone (LHRH), mixed meal, postural, metoclopramide, and vasopressin tests were performed in order to evaluate aberrant hormonal responses. Results were positive for vasopressin and upright posture test. Computed tomography (CT) imaging of her adrenal glands revealed bilateral multiple lobular masses (Fig. 1a, b); magnetic resonance imaging (MRI) of her orbits indicated bilateral exophthalmos with hypertrophy of the retro-orbital fat (Fig. 1c); MRI of her pituitary was normal (Fig. 1d). Diagnosis of PBMAH was made and our patient underwent bilateral laparoscopic adrenalectomy. Gross pathological examination of adrenal glands showed each gland contained several nodules (Fig. 1e). Pathological findings were consistent with PBMAH: multinodular glands with homogenous, golden-yellow-colored nodules. Nodules contained predominantly fascicular cells (Fig. 1f). After surgery, her plasma cortisol and ACTH at 8 a.m. were 6.06 ug/L and 1.87 ng/L respectively, indicating a successful surgery. Hydrocortisone supplementation was given as well, as she was started on metformin for diabetes. At 6-month follow-up, her blood glucose levels were well controlled and her blood pressure was 120/80 mmHg without use of any antihypertensive medication. The exophthalmos improved markedly after the surgery.

**Fig. 2:**
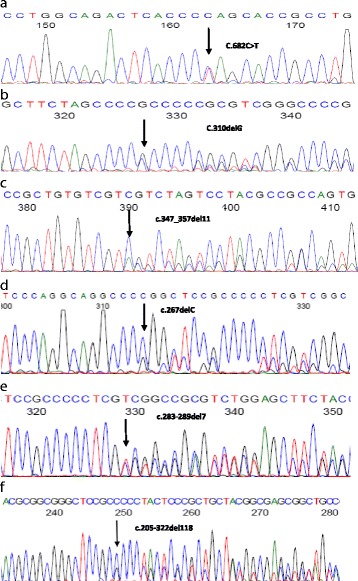
Germline and five somatic mutations in the *ARMC5* gene found in the patient with primary bilateral macronodular adrenal hyperplasia. Heterozygous germline mutation C.682C>T was present in all analyzed tissue (**a**). Heterozygous somatic mutations were found in five different nodules: C.310delG (**b**), c.347_357del11 (**c**), c.267delC (*arrow*) (**d**), c.283-289del7 (**e**), and c.205-322del118 (**f**)

After obtaining informed consent from our patient, DNA was extracted from peripheral blood leukocytes and seven different adrenal nodules that were obtained during surgery. All the coding and flanking intronic sequences of *ARMC5* were amplified by polymerase chain reaction (PCR). Mutations were identified by direct sequencing of PCR products. For the variations nomenclature, the main frequent isoform in the literature (NM_001105247.1) was used. We identified a heterozygous nonsense *ARMC5* germline mutation c.682C>T (p. Gln228*) in exon 3, which was predicted to change amino acid glutamine at position 228 to a stop codon (Fig. 2a). We also identified five heterozygous frameshift mutations (c.310delG, c.347_357del11, c.267delC, c.283_289del7, and c.205-322del118) in five different adrenal nodules (Fig. 2b–f). These mutations are predicted to cause a downstream stop codon with premature termination of translation (p.Ala104Profs*33, p.Ser116Tyrfs*6, p.Pro89Profs*48, p.Ser95Argfs*40, and p.Pro69Alafs*29 respectively). We did not find *ARMC5* mutations in the remaining two adrenal nodules. All the mutations are novel and not found in available online databases. Screening in first-degree relatives of our patient revealed no carriers of an *ARMC5* mutation.

**Fig. 3:**
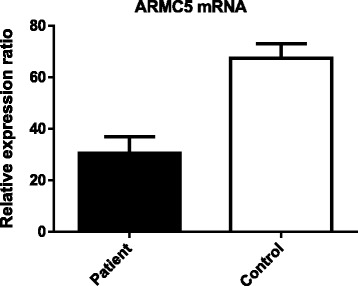
The messenger ribonucleic acid expression of *ARMC5* in the adrenal tumor samples of the patient and normal adrenal cortex. The *ARMC5* messenger RNA expression was lower in patient’s adrenal tumor samples compared to normal adrenal cortex. Patient is represented by *black bar* and control sample by *white bar*

Total ribonucleic acid (RNA) from the adrenal tumor tissue and two specimens of normal adrenal cortex were extracted and were reverse transcribed using a cDNA synthesis kit. To test whether the *ARMC5* mutation induced messenger RNA (mRNA) decay, real-time reverse transcriptase (RT)-PCR was performed. The *ARMC5* cDNA sequence was amplified with specific primers (5′-CTGGAGTGCAGTGACACGAT-3′ and 5′-TATCTGGGCATGGTGGTACA-3′). The relative *ARMC5* mRNA expression was determined by using the 2−ΔΔCT method and was normalized against the expression of the β-actin gene. The qRT-PCR analysis indicated the *ARMC5* mRNA expression was lower in our patient’s adrenal tumor samples compared to normal adrenal cortex (Fig. 3).

## Discussion

In our study, we present a case of a 52-year-old woman with a history of long-standing exophthalmos which was thought to be Graves’ ophthalmopathy but persisted despite treatment and she was later diagnosed as having Cushing’s syndrome due to primary bilateral macronodular hyperplasia. There have been only a few reports on atypical symptoms of Cushing’s syndrome and this report highlights the importance of exophthalmos and its association with hypercortisolism.

PBMAH is responsible for less than 1% of endogenous Cushing’s syndrome; quite often it has been found incidentally [3]. These tumors usually have low secretory capacity and result in subclinical Cushing’s syndrome over an extended period of time, leading to delay in diagnosis in a majority of patients. Although exophthalmos is typically associated with Graves’ disease, it has also been described in Cushing’s syndrome. As estimated [9], exophthalmos was present in 45% of active Cushing’s syndrome, 21% of iatrogenic Cushing’s syndrome, and 20% of treated Cushing’s syndrome in comparison to 2% in controls. The exact mechanism behind exophthalmos remains to be identified. It has been proposed that this is part of generalized fat redistribution typically seen in hypercortisolism. The excessive fat deposition in the orbit spares the orbital muscles. Some suggest increased lipoprotein lipase activity or increased glucocorticoid receptor density may be the cause behind this phenomenon [10–12]. Exophthalmos is an often forgotten clinical sign of Cushing’s syndrome at this time. In our study, we presented a case of PBMAH who only had the exophthalmos as a major symptom at the beginning and was treated as Graves’ ophthalmopathy until the classic features of Cushing’s syndrome appeared. This highlights the importance of early recognition of atypical symptoms of Cushing’s syndrome such as exophthalmos, which would save the patient from harmful effects of excessive cortisol exposure for an extended period of time and may help prevent the complications as well.

Recently, inactivating *ARMC5* mutations have been identified as a common genetic cause of PBMAH [3]. In our cohort, we identified a novel germline mutation c.682C>T and five novel somatic mutations in five different adrenocortical nodules which was in agreement with previous studies that most of the mutations observed were private and only a minority of them recurred [13, 14]. Although rearrangements or larger genomic deletion cannot be excluded in the remaining two nodules, the finding indicated that not every nodule carried another *ARMC5* variant [14]. Thus far, more than 30 germline and somatic pathogenic mutations have been reported [3–8, 14, 15]. Nonsense and frameshift mutations leading to nonsense transcripts contribute to nearly 60% of all pathogenic variants. It is known that mutations introducing premature translation stop codons (PTCs) trigger the mRNA surveillance pathway and nonsense-mediated decay [16]. In our study, the nonsense germline mutation and five frameshift somatic mutations all resulted in a premature stop codon and the corresponding mRNA could thus possibly be subjected to degradation through the nonsense-mediated decay. This hypothesis was supported by the fact that the total *ARMC5* mRNA expression was relatively low in our patient compared to control adrenal cortex. The exact function of ARMC5 protein and its associated signaling pathway are still largely unknown. The *in vitro* studies showed that transfecting the wild-type *ARMC5* gene induced apoptosis, while introducing *ARMC5* missense mutations abolished this effect [3, 17]. Thus, *ARMC5* inactivation leads to resistance to apoptosis in adrenocortical cells, which causes adrenal hyperplasia leading to increased cortisol level. Phenotype–genotype relations studies showed that patients with the *ARMC5*-damaging mutation exhibited a more severe hypercortisolism and larger adrenals compared to patients with the wild-type [13, 17]. Some reports also showed that *ARMC5* mutations are associated with specific patterns of illegitimate receptors expression. In our study, we noted abnormal responses after upright posture and vasopressin administration, which is in agreement with previous observations [3, 18].

## Conclusions

We demonstrated extensive genetic diversity of *ARMC5* in a patient with PBMAH that started with exophthalmos, which further expands the molecular pathophysiology of this disease. Early recognition of atypical symptoms and screening for *ARMC5* mutation in patients with PBMAH has important clinical implications for the diagnosis and genetic counseling.

## Abbreviations

ACTH: Adrenocorticotropic hormone
AIMAH: ACTH-independent macronodular adrenal hyperplasia
*ARMC5*: Armadillo repeat containing 5
BP: Blood pressure
CT: Computed tomography
H: Height
HbA1c: Glycated hemoglobin
LHRH: Luteinizing hormone-releasing hormone
mRNA: Messenger RNA
P: Pulse
PBMAH: Primary bilateral macronodular adrenal hyperplasia
PCR: Polymerase chain reaction
PTC: Premature translation stop codon
R: Respiration rate
RT: Reverse transcriptase
T: Temperature
TGAb: Anti-thyroglobulin antibodies
TPOAb: Anti-thyroid peroxidase
TRAb: Anti-thyrotropin receptor antibody
UFC: Urinary free cortisol
W: Weight
